# Effect of *Metarhizium anisopliae* on the fertility and fecundity of two species of fruit flies and horizontal transmission of mycotic infection

**DOI:** 10.1093/jis/14.1.100

**Published:** 2014-07-22

**Authors:** P. Sookar, s. Bhagwant, M.N. Allymamod

**Affiliations:** 1 Entomology Division, Agricultural Services, Ministry of Agro Industry & Food Security, Republic of Mauritius; 2 Faculty of Science, University of Mauritius, Republic of Mauritius

**Keywords:** biological control, mating behavior, entomopathogenic fungus

## Abstract

In Mauritius, the peach fruit fly, *Bactrocera zonata* Saunders (Diptera: Tephritidae), and the melon fly, *Bactrocera cucurbitae* (Coquillett), are the major pest of fruits and vegetables, respectively. Fruit growers make use of broad-spectrum insecticides to protect their crops from fruit fly attack. This method of fruit fly control is hazardous to the environment and is a threat to beneficial insects. The entomopathogenic fungus, *Metarhizium anisopliae* (Metchnikoff) Sorokin (Hypocreales: Clavicipitaceae), which was isolated from the soils of Mauritius, was used to investigate whether fungus-treated adult fruit flies could transfer conidia to non-treated flies during mating, and whether fungal infection could have an effect on mating behavior, fecundity, and fertility of the two female fruit fly species. When treated male flies were maintained together with non-treated female flies, they were able to transmit infection to untreated females, resulting in high mortalities. Similarly, fungus-infected female flies mixed with untreated males also transmitted infections to males, also resulting in high mortalities. Infection by *M. anisopliae* also resulted in the reduction of the number of eggs produced by females of *B. cucurbitae.* The results suggest that *M anisopliae* may have potential for use in integrated control programs of *B. zonata* and *B. cucurbitae* using the sterile insect technique in Mauritius.

## Introduction

The fruit flies *Bactrocera zonata* (Saunders) (Diptera: Tephritidae) and *B. cucurbitae* (Coquillett) are major pests limiting the production of fruits and cucurbits, respectively, in Mauritius ( Sookar et al. 2006 ). Fruit fly control strategies may include the use of chemical insecticides in bait spray for adult control, soil treatment with insecticides be neath host trees to kill fly larvae and puparia, male annihilation, use of biological control parasitoids), orchard sanitation, and application of sterile insect technique (SIT) ( Roessler 1989 ; Koyama et al. 2004 ; Vargas et al. 2004 ; kesi and Billah 2006). Although aerial or round application of insecticide-bait sprays as been effective, it is frequently regarded as serious threat to the environment ( Moreno nd Mangan 2000 ). Hence, the use of microbialcontrolagents,includingentomopathogenic fungi, as alternatives tochemical control and as a component of integrated pest management strategies is beingwidely explored for management of a widerange of fruit fly pests ( Dimbi et al. 2004 , 2009; Mochi Dinalva et al. 2006 ; Quesada-Moraga et al. 2006 ; Toledo et al. 2007 ; Daniel and Wyss 2009 ; Novelo-Rincón et al. 2009 ).

The control of crop pests with entomopathogenic fungi involves their application as inundative or augmentative releases as chemical pesticides using a device ( Vega et al. 2000 ). A complex set of interacting processes, both environmental and biotic, is necessary for or inhibitory to development of epizootics caused by entomopathogenic fungi. These include sensitivity to solar radiation ( Inglis et al. 1993 ; Alves et al. 1998 ); microbial antagonis-tics; host behavior, physiological condition, and age; pathogen vigour and age ( Moore et al. 1995 ); presence of pesticides ( Inglis et al. 2001 ); and appropriate temperature, humidity,and antagonists in the soil or on the host cuticle ( Moore and Prior 1993 ). Autodissemination is regarded as an appropriate strategy to increase the persistence of entomopathogenic fungi in the environment. This strategy uses target pests as carriers to selectively disseminate entomopathogens among their populations ( Maniania 1998 , 2002; Meadow et al. 2000 ; Rath 2000 )

However, fundamental to this approach is the efficient horizontal transmission of the pathogen to the target individuals within the pest population ( Furlong and Pell 2001 ). Successful transmission of *Metarhizium anisopliae* (Metchnikoff) Sorokin (Hypocreales: Clavicipitaceae) by honeybees for infection of the pollen beetle *Meligethes aeneus* F. (Coleoptera: Nitidulidae) ( Butt et al. 1998 ), of *Beauveria bassiana* (Balsamo) (Hypocreales: Clavicipitaceae) between adult flies of *Delia radicum* (L.) (Diptera: Anthomyiidae) ( Meadow et al. 2000 ), of *M. anisopliae* and *B. bassiana* between adult tsetse flies, *Glossina morsitans morsitans* (Diptera: Glossinidae) ( Kaaya and Okech 1990 ), and of *B. bassiana* between *Ips typographus* (L.) (Coleoptera: Scolitidae) ( Kreutz et al. 2004 ) confirms the capability of insects to transmit fungi horizontally.

Previous studies showed that Mauritian isolates of the entomopathogenic fungi, namely *Beauveria bassiana* (Balsamo) and *Metarhizium anisopliae* (Metchnikoff) Sorokin, are pathogenic to fruit flies ( Sookar et al. 2008 and 2010). The locally isolated fungi could be used in an SIT-pathogen approach. The objectives of this study were, therefore, to investigate whether *M. anisopliae* -treated adult fruit flies could transfer conidia to non-treated flies during mating and to study the effects of fungal infection on mating behavior, fecundity, and fertility of two female fruit fly species.

## Materials and Methods

### Insects

The laboratory-reared *B. zonata* and *B. cucur bitae* used in the study were maintained for∽140 and 44 generations, respectively, at the Entomology Division of the Ministry of Agro Industry and Food Security at Reduit, Republic of Mauritius. Approximately 400 adults (both males and females) were kept in Plexiglas cages (30 cm × 30 cm × 30 cm) and provided with water and a 3:1 volumetric mixture of sugar and enzymatic yeast hydrolysate (United States Biochemical, Cleveland, OH). As soon as reproductive maturity was attained (12 days after adult emergence), an egging device, consisting of a plastic cylindrical tube with a volume of 30 cm ^3^ and perforated with evenly spaced holes of 1 mm diameter, was used for oviposition. A dark cloth (5 × 5 cm) was saturated with guava juice and placed inside the egging device. The egging device was placed inside the cage for a period of 24 hours every week. The collected eggs were transferred to an artificial diet composed of sugarcane bagasse (6.0%), ground maize (6.0%), sugarcane (11.0%), waste brewer’s yeast (6.0%), wheat bran (6.0%), benzoic acid (0.1%), nipagen (0.1%), and water (64.8%) at the rate of 0.8 mL eggs/kg diet. ( Sookar et al., in press ). The trays with developing larvae were kept at 25 ± 1°C and 60–70% RH. Third-instar larvae emerged and pupated in vermiculite. Pupae were collected for a period of five days, sieved, and then kept in a dark room at 25 ± 1°C and 60–70% RH for about seven days before being transferred to adult cages.

Adults of *B. zonata* and *B. cucurbitae* were obtained from the mass rearing stock maintained at the Entomology Division, Ministryof Agro Industry & Food Security. Two-week-old flies were used in this experiment.

### Fungal isolate

*Metarhizium anisopliae* , previously reported to be pathogenic to the two species of fruit flies ( Sookar et al. 2008 ), was used in this study. The fungus was isolated by soil baiting with waxworm larvae ( *Galleria mellonella* L.(Lepidoptera: Pyralidae)), and it was deposited at the International Centre for Insect Physiology and Ecology in Nairobi, Kenya. The isolate was grown on Sabouraud dextrose agar (SDA) in 90 mm Petri dishes and maintained at ambient temperature (22–28°C) incomplete darkness. Conidia were harvested by scraping the surface of a three-week-old culture. Spores were suspended in 20 mL sterile istilled water containing 0.05% Triton X-100 in glass bottles containing 3 mm glass beads. Bottles were stoppered and vortexed for 5 min to produce a homogeneous conidial suspension. Conidia were then quantified with a haemocytometer following serial dilution insterile distilled water. The viability of conidia was determined by spread-plating 0.1 mL of conidial suspension (titrated to 3x106 conidiaml-1) on four SDA plates. Sterile microscope cover slips were placed on each plate. The plates were incubated at 24–29ºC and examined after 20 hr. Percentage germination was determined by counting approximately 100 spores for each plate at 200× magnification.Each plate served as a replicate with four replicationsper isolate.

### Horizontal transmission of fungus

Four groups each of 10 two-week-old male B. *zonata* and B. *cucurbitae* were contaminated with dry conidia of M. *anisopliae* through a velvet material covering the inner side of a cylindrical plastic tube (95 × 50 mm) that had the bottom removed. Dry conidia (0.3 g) were spread evenly onto the velvet material. Four groups of 10 two-week-old male *B. zonata* and *B. cucurbitae* were transferred to the cylindrical tube (which was then covered with the lid) and allowed to walk on the velvet for two minutes. The flies were then released into 300 ×300 × 300 plastic cages. An equal number of seven-day-old uninfected female flies of the same species were released into the cages after one day. Insects were maintained together for 24 hr to allow time for mating. A group of 20 untreated males and females were also held together for 24 hr as controls. Flies were then separated by sex and held in separate cages for 15 days at room temperature, and mortalities in both sexes were recorded. The experiment was replicated four times. In another experiment, four groups of two-week-old female *B. zonata* and *B. cucurbitae* were contaminated with fungal conidia and mixed with equal numbers of seven-day-old untreated and unmated males after one day. The experimental procedures remained the same as described above.

To determine the number of healthy flies to which a treated fly could pass infection, a single sexually mature female fly (12 days old) was exposed to dry conidia of *M. anisopliae* and served as a “donor.” The latter was placed in a small cage made from a cylindrical plastic tube (150 mm × 40 mm diam.) with three untreated sexually mature males (12 days old) that served as “recipients.” Flies were then separated after 24 hr and transferred individually to the small cages. Three additional sexually mature male flies were introduced every day for five days into a clean cage containing a donor female fly. The male flies were transferred individually to clean cages each time the pairs separated and were maintained at ambient conditions. Mortality was recorded for 15 days. Flies were not treated with fungal conidia in the control treatment, but the experimental procedure remained the same. Flies were maintained on a sugar-yeast diet, and a sponge soaked in water was added to cages as the water source. The experiment was repeated four times. A similar experiment was carried out with a sexually mature male fly as donor and sexually mature female flies as recipients in the same conditions as described above. This experiment was also replicated four times. All male and female flies that were used in the tests were separated by sex immediately following eclosion.

In all cases, the dead flies were surface sterilized with 70% alcohol, rinsed three times in sterile distilled water, and then placed on moist filter paper and incubated at room temperature. Fungal growth on the insect surface confirmed death was due to mycosis.

### Effect of fungal infection by *M. anisopliae* on the fecundity and fertility of female fruit flies

Ten females (12 days old) of each species were exposed to conidia of *M. anisopliae* as described above in order to assess the effect of fungal infection on the fertility of *B. cucurbitae.* Treated female flies were placed in Plexiglas cages (150 × 150 × 240 mm). An equal number of male flies (12 days old) of the same species were added to each cage and maintained together until all treated females died. The flies were supplied with a sugar-enzymatic yeast hydrolysate diet and water. A plastic container (diameter 30 mm and height 65 mm) with holes of 1 mm diameter (one hole/cm ^2^ ) was used as an oviposition apparatus. A sponge soaked in 10% honey solution was placed inside the plastic container as an oviposition stimulant. The eggs were collected by washing the container with water. The number of eggs was counted daily. Female mortality was also recorded every day. The same experiment was repeated with treated male flies.

To evaluate the effect of fungal infection on fertility, 20 eggs from each treatment and replicate were chosen at random each day and transferred to a damp black cloth in a 90 mm Petri dish. Plates were incubated at room temperature and the number of eggs that hatched was recorded after 72 hours.

### Data analysis

In all tests, fly mortality due to fungal infection was adjusted for natural mortality in the controls using Abbott’s formula (1925) . Data were analysed using the ANOVA procedure of SAS ( SAS Institute 2001 ). Tukey’s test was used to compare mortalities and fecundity at an alpha level of 0.05.

## Results

### Horizontal transmission of fungal conidia during mating

In viability tests, the average germination of conidia was 94% after 20 hr on SDA plates. The number of conidia picked up by donor flies varied between 2.9 × 10 ^5^ and 1.0 × 10 ^6^ , while recipient flies picked up 1 × 10 ^5^ to 2.7 × 10 ^5^ conidia. Mortality in the controls did not exceed 5%. Male and female donors of *B. zonata* and *B. cucurbitae* exposed directly to conidia of *M. anisopliae* became infected and all died of fungal infection within six to seven days postexposure. When treated male (donor) flies were maintained together with non-treated female (recipient) flies, they were able to transmit infection to untreated females, resulting in mortalities of 83 ± 5% in *B. zonata* and 69 ± 5% in *B. cucurbitae* 14 days post-inoculation. Similarly, fungus-infected female donor flies mixed with untreated males also transmitted infections to male recipients, resulting in mortalities of 88 ± 6% in *B. zonata* and 78 ± 4% in *B. cucurbitae* 13 days post-inoculation. Male and female donors of *B. zonata* and *B. cucurbitae* exposed directly to conidia of *M. anisopliae* became infected and all died of fungal infection within six to seven days postexposure.

**Figure 1.**
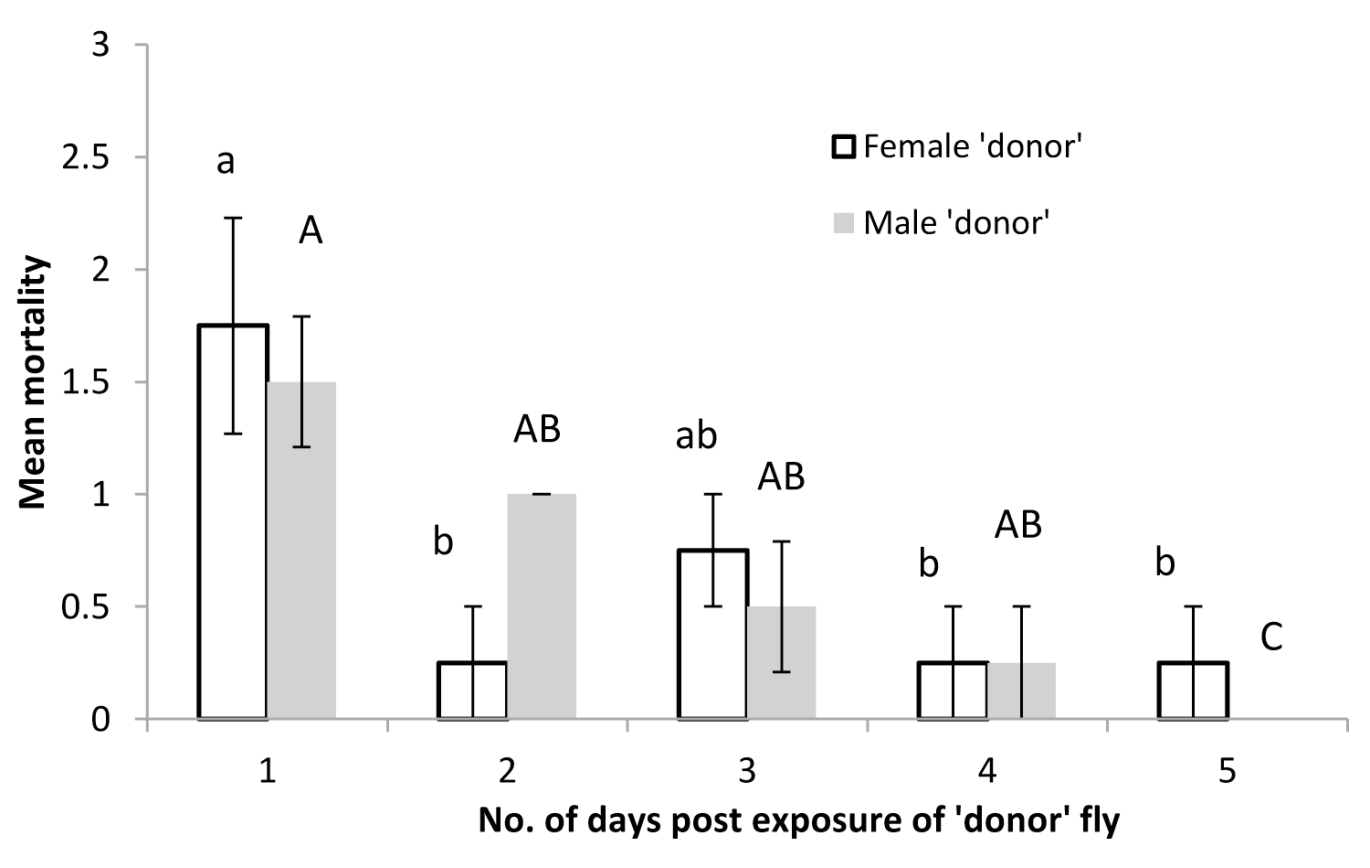
Mean mortality (±SEM) of *Bactrocera zonata* due to horizontal transmission of *Metarhizium anisopliae* from one treated fly (donor) to three uninfected flies (recipients) of the opposite sex at 1, 2, 3, 4, and 5 days postexposure to the donor fly. Each day on the x-axis represents different batches of successively introduced flies, and each column represents mean mortality of three flies exposed to one donor fly. Treatment female donor (unshaded bars): histograms with the same small letter are not significantly different (ANOVA and Tukey’s test, *P* < 0.019). Treatment male donor (shaded bars): histograms with the same capital letter are not significantly different (ANOVA and Tukey’s test, *P* < 0.001). High quality figures are available online.

**Figure 2.**
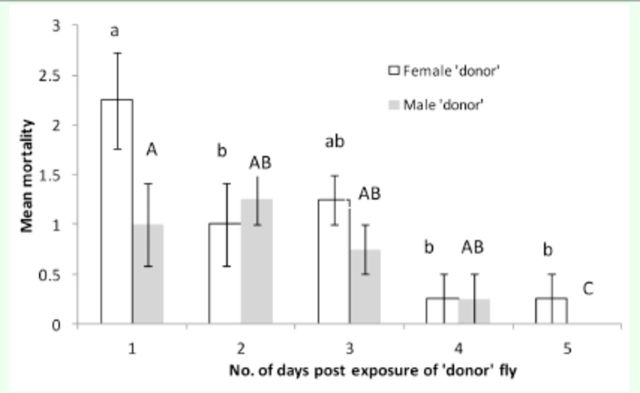
Mean mortality (±SEM) of *Bactrocera cucurbitae* due to horizontal transmission of *Metarhizium anisopliae* from one treated fly (donor) to three uninfected flies (recipients) of the opposite sex at 1, 2, 3, 4, and 5 days postexposure to the donor fly. Each day on the x-axis represents different batches of successively introduced flies and each column represents mean mortality of three flies exposed to one donor fly. Treatment female donor (unshaded bars): histograms with the same small letter are not significantly different (ANOVA and Tukey’s test, *P* < 0.005). Treatment male donor (shaded bars): histograms with the same capital letter are not significantly different (ANOVA and Tukey’s test, *P* < 0.015). High quality figures are available online.

There was no significant difference in mortality of recipient flies exposed to either a female donor or a male donor for both *B. zonata* (F _1, 39_ = 0.023, *P* = 0.881) ( Figure 1 ) and *B. cucurbitae* (F _1,__39_ = 1.239, *P* = 0.273) ( Figure 2 ). Mortality of recipient flies (males and females of *B. zonata* or *B. cucurbitae* ) was significantly affected by the day (Day 1, 2, 3, 4, or 5) on which they were exposed to the treated donor fly. Furthermore, mortality had a strong negative correlation to the number of days (1, 2, 3, 4, and 5 days) postexposure to the treated donor fly ( Table 1 ).

**Table 1.**
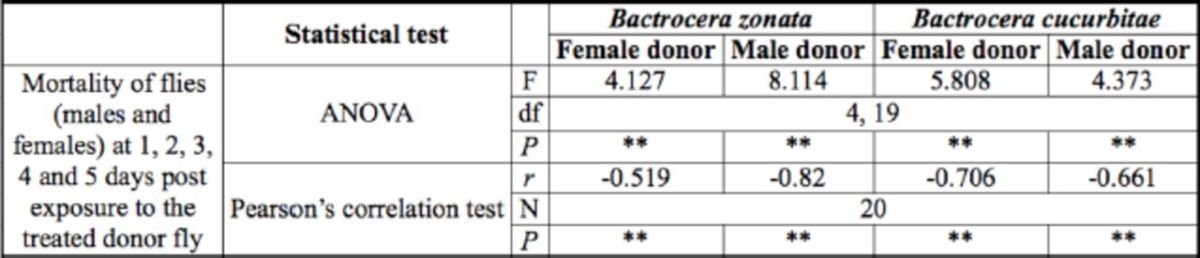
One-way ANOVA and Pearson’s correlation of mortal ity of flies (males or females) at 1, 2, 3, 4, and 5 days postexposure to *Metarhizium anisopliae-treated* donor fly (male or fem ale) of *Bactrocera zonata* and *B. cucurbitae.*

*r =* Pearson correlation coefficient; *, *P* < 0.05; **, *P* < 0.01

### Fungal infection by *M. anisopliae* on the fecundity and fertility of female *B. cucurbitae*

Mortality in the controls did not exceed 5%. Male and female donors of *B. cucurbitae* exposed directly to conidia of *M. anisopliae* became infected and all died of fungal infection within six to seven days postexposure.

**Figure 3.**
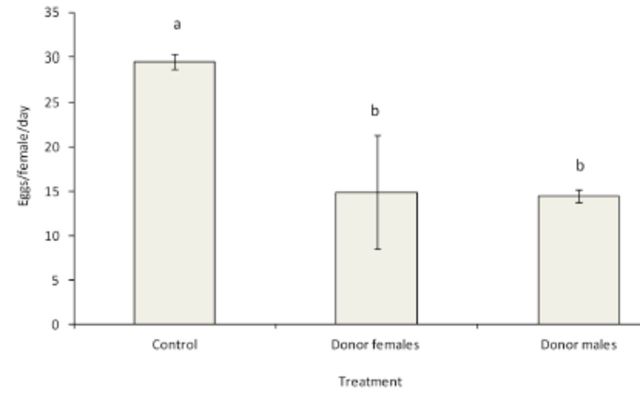
Mean number of eggs/female/day (±SEM) of *Bactrocera cucurbitae* (N = 10) for a period of seven days after exposure to *Metarhizium anisopliae.* Histograms with the same letter are not significantly different (ANOVA and Tukey’s test, *P* < 0.0001). High quality figures are available online.

Treatment of either the females or the males of *B. cucurbitae* with *M. anisopliae* resulted in significantly lower fecundity ( *F*_2,__42_ = 10.941, *P* < 0.0001) as compared to the untreated flies ( Figure 3 ). However, there was no significant difference in fecundity ( *P* > 0.05) between *B. cucurbitae* donor females and donor males.

Fertility, expressed as the percentage of hatched eggs, was not affected by fungal infection. Mean egg hatch for treated females, recipient females, and untreated females of *B. cucurbitae* was 53 ± 6, 60 ± 5, and 55 ± 6, respectively. (F _1,__2_ = 0.16, *P* = 0.8499).

## Discussion

The success of fungi as biocontrol agents would depend on their dispersal efficiency and their ability to cause acute mortality in the host population. When fungus-treated fruit flies of both sexes were mixed with healthy flies of opposite sexes, they were capable of transmitting fungal conidia to the healthy ones, causing mortality of up to 88% 14 days after inoculation. It was also demonstrated in this study that a contaminated fly is able to transfer a fatal dose of inoculum to at least three mates before it dies of fungal infection. Several studies have reported horizontal transmission of fungal infection from infected insects to healthy ones through mating or physical contact in different insect orders ( Kaaya and Okech 1990 ; Maniania 1994 ; Milner and Staples 1996 ; Kaakeh et al. 1996 ; Hedström and Monge-Nájera 1998 ; Roy and Pell 2000 ; Meadow et al. 2000 ; Dimbi 2003 ). Kaaya and Okech (1990) reported that adult tsetse fly, *Glossina morsitans morsitans* Westwood, treated with *M. anisopliae* and *B. bassiana* and mixed with an equal number of healthy flies were able to transmit the disease during mating and physical contact. Maniania (1994) reported that a single *Metarhizium-* infected *Glossina morsitans centralis* Machdo could spread the disease to five to 10 healthy flies under laboratory conditions. Meadow et al. (2000) demonstrated that cabbage root flies, *Delia radicum* L., were able to receive and transmit fatal doses of inoculum to at least six flies in a chain.

According to Hedström and Monge-Nájera (1998) , because fungal infection requires contact between sexes, infected females or males that copulate more than once increase the chances of transfer of disease within populations. The fact that, in this experiment, flies were observed to mate more than once is likely to increase the chances for an infected fly to transmit infection to several mates before it dies. In the field, male-to-male transmission could also be enhanced when male flies aggregate during leks, as in the case of the Mediterranean fruit fly ( Shelly and Villalobos 1995 ; Lance et al. 2000 ).

Our results show that infection by *M. anisopliae* did not only result in mortality of fruit flies, but also resulted in the reduction of the number of eggs produced by females of *B. cucurbitae* from 30 ± 1 eggs/untreated female to 15 ± 6 eggs/treated female. Castillo et al. (2000) obtained 65% reduction in fecundity of the Mediterranean fruit fly treated with *P. fumosoroseus* and 40-50% reduction with *M. anisopliae* and *A ochraceus.*Eilenberg (1987) reported that infection by *Entomophthora muscae* reduced fecundity in some Diptera, such as the onion maggot, *Delia antiqua* (Meigen); the carrot fly, *Psila rosae* Fabricius; and the house fly, *Musca domestica* L. However, no significant difference in the hatchability of eggs was observed between the eggs from treated flies and the untreated controls. Those findings corroborate the results obtained in this study.

Toledo et al. (2007) evaluated the mating performance of adult *Anastrepha ludens* (Loew) treated with two products of the fungus *B. bassiana* (LCPP and Bassianil) in field cage conditions. Horizontal transmission to females during the first day was 80.6% and 84.3% through mating and 15.4% and 21.6% through attempts to mate and contact during courtship for the LCPP and Bassianil products, respectively. Egg hatch was not significantly affected by fungal infection. Lund and Hajek (2005) reported that *M. anisopliae-treated* Asian longhorned beetle, *Anoplophora glabripennis* (Motschulsky), had shorter longevity (10.6 days versus 25.8 days) *,* lower fecundity (4.4 eggs/female versus 13.0 eggs/female), and fewer eggs hatch (70.2% versus 77.8%) as compared to untreated females.

It has been shown in this study that a single treated fly of either *B. zonata* or *B. cucurbitae* (male or female) could pass infection to at least three healthy flies before it died. In the implementation of the sterile insect technique (SIT), there are inundative releases of sterile insects to reduce reproduction of the same species. When a sterile male mates with a wild female, the latter lay infertile eggs. After mating with a sterile male, a wild female may mate with a wild male and produce fertile eggs. On the other hand, a fungus-treated sterile male could transfer a lethal dose of conidia to wild females through mating. Furthermore, in cases where a bisexual strain is used in an SIT program, the released sterile females could be used as vectors of the entomopathogenic fungus so as to transmit mycotic infection to wild males through mating.

In conclusion, this study has demonstrated that horizontal transmission of fungal infection does occur during mating and physical contacts. Fungus-infected female flies laid fewer eggs than healthy flies, but the infection did not have any effect on the fertility of the eggs. These results suggest that *M. anisopliae* could be incorporated into SIT for the control of *B. zonata* and *B. cucurbitae* in Mauritius. However, the effect of *M. anisopliae* on the performance of the sterile insects needs to be investigated. The effect of the fungal inoculum on the sterile insects with regard to their ability to fly, avoid predators, find leks, court, and compete with wild males for mating should be determined before embarking on an SIT-pathogen approach.
